# Recurrent DKA results in high societal costs – a retrospective study identifying social predictors of recurrence for potential future intervention

**DOI:** 10.1186/s40842-021-00127-6

**Published:** 2021-08-01

**Authors:** Ryan Lyerla, Brianna Johnson-Rabbett, Almoutaz Shakally, Rekha Magar, Hind Alameddine, Lisa Fish

**Affiliations:** 1 Allina Health, Minneapolis, MN USA; 2 University of Nebraska Medical Center, Omaha, NE USA; 3 Socal Endocrinology, Chino, CA USA; 4 HealthPartners, Anoka, MN USA; 5 Hennepin Health, Minneapolis, MN USA

**Keywords:** Recurrent DKA, Insulin cost, Barriers to care

## Abstract

**Aims:**

Diabetic ketoacidosis (DKA) is an emergency with high morbidity and mortality. This study examined patient factors associated with hospitalization for recurrent DKA.

**Methods:**

Characteristics of 265 subjects admitted for DKA at Hennepin County Medical Center between January 2017 and January 2019 were retrospectively analyzed. Differences between subjects with a single admission versus multiple were reviewed.

**Results:**

Forty-eight out of 265 patients had recurrent DKA. Risk factors included African American race (adjusted odds ratio (aOR) versus white non-Hispanic = 4.6, 95% CI 1.8–13, *p* = 0.001) or other race/ethnicity (aOR = 8.6, 2.9–28, *p* < 0.0001), younger age (aOR 37-52y versus 18-36y = 0.48, 0.19–1.16, *p* = 0.10; aOR 53-99y versus 18-36y = 0.37, 0.12–0.99, *p* = 0.05), type 1 diabetes mellitus (aOR = 2.4, 1.1–5.5, *p* = 0.04), ever homeless (aOR = 2.5, 1.1–5.4, *p* = 0.03), and drug abuse (aOR = 3.2, 1.3–7.8, *p* = 0.009). DKA cost a median of $29,981 per admission.

**Conclusions:**

Recurrent DKA is costly, and social determinants are strong predictors of recurrence. This study highlights the need for targeted preventative care programs.

## Background

Diabetic ketoacidosis (DKA) is a dangerous complication of type 1 and type 2 diabetes mellitus with high morbidity [1]. It results in prolonged hospital stays that include mortality risk, while incurring high healthcare costs [2, 3]. Notably, data from the CDC’s United States Diabetes Surveillance System identified an increase in rates of hospitalization for DKA during the years of 2009–2014 [2]. Precipitants of DKA include infections and non-compliance with insulin. Non-compliance is associated with younger age, drug and alcohol abuse, psychiatric comorbidity, socioeconomic status, homelessness, and fragmentation of care [4–7]. A recent German study specifically identified a dose dependent association between alcohol use and DKA occurrence in youths with type 1 diabetes mellitus [8]. One recent case–control study comparing patients with single episodes of DKA vs recurrent DKA found only minimal differences in the two groups, indicating that further study is needed to help identify factors that contribute to recurrent DKA [9].

Hennepin County Medical Center (HCMC) is a teaching hospital in Minneapolis, Minnesota that serves a diverse and underserved inner-city population with high rates of recurrent DKA. The purpose of this study was to retrospectively analyze patient factors that may contribute to recurrent DKA.

## Methods

We conducted an observational retrospective case–control study comparing patients with diabetes mellitus who had one versus more than one (defined as recurrent) DKA admission to Hennepin County Medical Center (HCMC) in Minneapolis, Minnesota between January 1, 2017 and January 1, 2019. HCMC services the central Minneapolis population and surrounding communities.

Inclusion criteria for this study were patients age > / = 18 with one or more admission that resulted in an ICD-10 discharge diagnosis code of E11.10 (DKA) between the dates of January 1, 2017 and January 1, 2019. DKA was defined as serum bicarbonate < 18 mg/dl, anion gap >  = 16, beta hydroxybutyrate > 3 mmol/l, and admission blood glucose of > 250 mg/dl. Patients who specifically requested to opt out from research in general for retrospective collection were excluded. 265 total patients were included in the analysis.

All patients discharged from HCMC are coded using standard internationally agreed upon codes. Data is stored and collected from an electronic health record. Repository data included demographic information (age, sex, race/ethnicity, insurance status defined as last status on file, living status), health information (diabetes type, hemoglobin A1c, admission point of care glucose), socioeconomic factors (homelessness), mental illness, and substance abuse (alcohol and drugs). Patients with inpatient DKA encounters were separated into two groups of 1 or >  = 2 DKA encounters during the study period of 2017–2019. A recurrent patient was defined as a patient who had more than one hospitalization for DKA during the study period of 2 years.

The Hennepin Health Board Research Review Committee granted ethics approval and research was performed in accordance with the Code of Ethics of the World Medical Association for experiments involving humans.

### Retrospective study size and statistical analysis

The sample size was determined by the number of people admitted with DKA fulfilling the inclusion criteria. Associations between patient characteristics and DKA recurrence were examined using penalized logistic regression models, including univariate models with a single predictor characteristic, and a parsimonious multivariate model with predictors chosen from the full set of candidate predictors by backward variable selection using a penalized likelihood ratio test [10]. Analyses were conducted using R version 3.5.2 including the logistf package version 1.23 [11, 12].

## Results

The clinical characteristics of the 265 subjects during the 2 year study period from January 1, 2017 to January 1, 2019 are shown in Table 1. A comparison between patients with single episode of DKA versus recurrent DKA are shown in Table 2.

**Table 1:** Clinical characteristics of the 265 patients with single or recurrent DKA

Gender	Male (N, %)	Female (N, %)		
	158 (60%)	107 (40%)		
Race	White (non-Hispanic) (N, %)	African American (N, %)	Other (N, %)	
	95 (36%)	119 (45%)	51 (19%)	
Age	Mean, Range, SD			
	45, 18–99, 15			
Insurance	Medicaid (N, %)	Medicare (N, %)	Commercial/other/self pay (N, %)	Unknown (N, %)
	143 (54%)	60 (23%)	36 (14%)	26 (10%)
Diabetes type	Type 1 (N, %)	Type 2 (N, %)		
	116 (44%)	149 (56%)		
Ever homeless	Yes (N, %)	No (N, %)		
	71 (27%)	194 (73%)		
Alcohol abuse	Yes (N, %)	No (N, %)		
	34 (13%)	231 (87%)		
Drug abuse	Yes (N, %)	No (N, %)		
	41 (15%)	224 (85%)		
History of psychosis	Yes (N, %)	No (N, %)		
	33 (12%)	232 (88%)		
Insulin pump use	Yes (N, %)	No (N, %)		
	12 (5%)	253 (95%)

**Table 2:** DKA single vs recurrent episodes

	DKA recurrence	Multivariate analysis	
	n, number of patients with DKA recurrence/N, total number of patients in category, (%)	OR, (95% CI)	*P*-value
Race			
White	7/95, (7%)	1, (-)	0.0001
African American	26/119, (22%)	4.63, (1.82–13.29)	
Other ethnicity	15/51 (29%)	8.55, (2.93–27.94)	
Age			
18-36y	27/89, (30%)	1, (-)	0.11
37-52y	14/89, (16%)	0.48, (0.19–1.16)	
53-99y	7/87, (8%)	0.37, (0.12–0.99)	
Type 1 DM			
No	17/149, (11%)	1, (-)	0.04
Yes	31/116, (27%)	2.39, (1.06–5.47)	
Ever homeless			
No	26/194, 13%)	1, (-)	0.03
Yes	22/71, (31%)	2.45, (1.11–5.44)	
Drug abuse			
No	31/224, (14%)	1, (-)	0.009
Yes	17/41, (41%)	3.22, (1.34–7.84)	
eGFR < 30 ml/hr			
No	42/247, (17%)	1, (-)	0.12
Yes	6/18, (33%)	2.62, (0.77–8.56)	

For the whole cohort of those with single or recurrent DKA, there were 119 (45%) African American subjects (compared to 34% for all hospital admissions at Hennepin County Medical Center).Lack of social support was common, psychiatric illnesses were prevalent with 79 patients (30%) with a history of depression and 33 patients (12%) with a history of psychosis which included conditions such as bipolar disorder, schizophrenia, and schizoaffective disorder.

Patients with a history of drug abuse were more likely to have recurrent DKA during the study period. Other significant predictors of DKA recurrence included African American race and other race/ethnicity, history of homelessness, type 1 diabetes, and younger age.

Other factors that tended to be associated with DKA recurrence but were not significant after adjusting for other factors included a history of depression (25% vs. 15%, unadjusted odds ratio (OR) = 1.9, 1.0–3.6, *p* = 0.05), low eGFR (33 vs. 17%, OR = 2.5, 0.87–6.7, *p* = 0.09), and insulin pump use (0 vs. 19%, OR = 0.17, 0.00–1.33, *p* = 0.11). Patients with a history of mental illness (depression, psychosis, or both) tended to have higher rates of DKA recurrence (24 vs. 15%, OR = 1.8, 0.98–3.5, *p* = 0.06). Eighteen (7%) patients in the retrospective analysis died during the 2 year study period, a rate of 5% (18/379) per hospitalization for DKA. Mortality rates did not differ between patients without and with recurrent DKA (6% (13/217) vs. 10% (5/48), OR = 1.9, 95% CI 0.62–5.2, *p* = 0.24).

Hospitalization cost data was collected. The median cost per admission for DKA was $29,981 with a range from $10,838 to $284,357. One patient who had 11 admissions for recurrent DKA in the 1-year study period accumulated charges of $476,831.

## Discussion

In this retrospective study of 265 patients with diabetes mellitus that experienced DKA, recurrence was predicted by many social determinants of health including psychiatric comorbidities, alcohol and drug abuse, homelessness, and non-white race It was notable that the mortality rate of DKA was high at 10% over a 2 year period in a group with a mean age of 32 years. In addition, financial costs of DKA were high at $29,981 per admission.

Diabetic ketoacidosis (DKA) remains a potentially devastating complication of both type 1 and type 2 diabetes. In recent decades, the health care system (both the United States and elsewhere) has been unable to reduce the overall frequency or improve the morbidity of the disorder. The CDC’s United States Diabetes Surveillance System (USDSS) found that in-hospital mortality rate declined from 1.1% in 2000 to 0.4% in 2014, however the rate of hospitalizations actually increased during this time period. During 2009–2014, the rate increased 54.9% from 19.5 to 30.2 per 1,000 persons, at an average annual rate of 6.3% [2]. This leaves many health care providers frustrated and searching for answers.

This study aimed to identify potential factors that increase the risk of recurrent DKA. Elucidating factors that impact recurrence may help us understand the barriers to achieving good diabetes control.

Insulin discontinuation or non-adherence is an important precipitating cause of DKA in many retrospective studies. In the study by Randall et al. (2011) examining 164 inner-city minority patients (96% African American) at Grady Hospital in Atlanta with DKA, 68% of patients developed DKA secondary to insulin non-adherence [6]. Poor compliance accounted for 56% of patients with first-time episodes and 78% of patients with recurrent episodes. While the largest percentage gave no reason for stopping, 27% reported lack of money to buy insulin and 5% reported stretching insulin due to cost. Insulin costs are soaring in the United States with prices doubling from 2012 to 2016. A patient with Type 1 diabetes mellitus incurred annual insulin costs of $5,705, on average, in 2016. The average cost was roughly half that, at $2,864 per patient, in 2012, according to a report by the nonprofit Health Care Cost Institute in January 2019 [13].

Despite rising costs of insulin, hospitalization for DKA remains exponentially more expensive. It is estimated that DKA episodes represent more than $1 of every $4 spent on direct medical care for adult patients with type 1 diabetes and $1 of every $2 in patients with multiple episodes of DKA [14]. This is confirmed by cost data collected in this retrospective study. With the median hospitalization cost of $29,981, this far outweighs even the most costly insulins on the market.

Cost is not the only barrier affecting insulin adherence. We attempted a pilot intervention supplying free long-acting insulin to patients who had more than 1 episode of DKA in the previous 2 years. We found that patients had multiple challenges with lack of consistent ability to receive communication due to intermittent cell phone access, lack of private transportation and lack of funds for public transportation, and difficulty with life situations that precluded making or keeping appointments. Looking at the barriers involved, we concluded that a program of insulin access would need to allow patients to obtain insulin from a local pharmacy and be accessible on a regular unscheduled basis. Acknowledging that this would need to be a county or state-wide program and would have significant expense, for this targeted group of patients it would save money due to their high hospital costs.

Housing insecurity and homelessness, psychiatric illness, and alcohol and drug abuse are additional barriers affecting insulin adherence. Randall et al. (2011) found that compared with patients with first-time DKA, those with recurrent DKA had higher rates of depression, alcohol use, drug abuse, and homelessness [6]. In our retrospective study, we identified an association between recurrent DKA and being younger, African American or other non-white race, ever experiencing homelessness, history of drug abuse, and type 1 diabetes. Psychiatric illness was prevalent among all patients who presented with DKA (36%) and recurrence rates tended to be higher in this group. Recently there has been a growing awareness of the inequality of social determinants of health (SDOH) leading to health disparities and poor DM control. Silva-Tinoco et al. conducted a multicenter cross-sectional study in patients with type 2 diabetes (T2D) from 28 primary care outpatient centers located in Mexico City and found that socioeconomic and educational gradients influence diabetes knowledge among primary care patients with type 2 diabetes [15]. Education and socioeconomic status are not the only SDOHs affecting DM care. Economic stability (employment, food security, housing, poverty, education), social & community context (social safety net, incarceration), neighborhood & physical environment (transportation, food availability, housing availability) and nearby health care availability are all important factors [16].

A major limitation of this study is that it is retrospective, and we are limited to information available in the medical record. We were not able to reliably identify important factors relating to employment/income status, family stressors including the need to care for small children or elderly relatives, periods of incarceration, emergency room use, and involvement of diabetes education providers. DKA as a complication of DM is very likely to be affected by those SDOHs and would be best addressed in a prospective study of recurrent DKA patients.

Another important aspect of the data is that patients admitted with DKA were 44% type 1 and 56% type 2. While DKA in type 2 diabetes is well described in the literature and is reported to involve patients of mainly black and Latino ethnicity [17], there are important aspects of clinical presentation, phenotypic features, and outcomes after therapy that need further exploration in future studies.

Our findings add to the body of literature demonstrating a multitude of factors associated with DKA recurrence. We examined a relatively large population of patients (265) from a county hospital over 2 years. Forty-eight of these patients (18%) had recurrent DKA during the study period.

## Conclusions

Important insight was gained studying this challenging population. This is a generally young to middle-aged group of people suffering from a time consuming and resource straining disease with high morbidity and mortality. There is a compelling need to increase awareness about this underserved patient population and work to reduce barriers to obtaining essential health care. Addressing and overcoming these barriers, which are key Social Determinants of Health, is critical in allowing patients with fragile social supports to obtain equitable care and health in our society. This may include government or community programs offering free or heavily discounted insulin (even $20 copays may be too high for those on a very limited income), public transportation, cell phone service, psychiatric care and drug counseling, stable housing, and home health nurse services. As demonstrated in this paper, the monetary cost of hospital admission for DKA is extremely high and a preventative approach would likely improve quality of life for these patients while also saving vast amounts of costs related to hospital admission and resulting in net savings in terms of overall monetary as well as social costs.

## Data Availability

The datasets used and/or analyzed during the current study are available from the corresponding author on reasonable request.

## Abbreviations

DKA: Diabetic ketoacidosis
aOR: Adjusted odds ratio
HCMC: Hennepin County Medical Center
SDOH: Social determinants of health
